# Differential allocation in a gift-giving spider: males adjust their reproductive investment in response to female condition

**DOI:** 10.1186/s12862-021-01870-1

**Published:** 2021-07-08

**Authors:** Diego Solano-Brenes, Luiz Ernesto Costa-Schmidt, Maria Jose Albo, Glauco Machado

**Affiliations:** 1 Programa de Pós-graduação em Ecologia, Instituto de Biociências, Universidade de São Paulo, São Paulo, Brazil; 2 Departamento de Ecologia, Zoologia e Genética, Instituto de Biologia, Universidade Federal de Pelotas, Campus Universitário do Capão do Leão, Pelotas, Rio Grande do Sul Brazil; 3 Departamento de Ecología y Biología Evolutiva, Facultad de Ciencias, Universidad de la República, Montevideo, Uruguay; 4 Departamento de Ecología y Biología Evolutiva, Instituto de Investigaciones Biológicas Clemente Estable, Montevideo, Uruguay; 5 LAGE do Departamento de Ecologia, Instituto de Biociências, Universidade de São Paulo, São Paulo, Brazil

**Keywords:** Body condition, Copulatory courtship, Cryptic male choice, Male mate choice, Mating effort, Parental effort, Pre-copulatory courtship, Sperm transfer

## Abstract

**Background:**

When males are selective, they can either reject low-quality females or adjust their reproductive investment in response to traits that indicate female quality (e.g., body size or condition). According to the *differential allocation hypothesis*, males increase their reproductive investment when paired with high-quality females (*positive differential allocation*) or increase their reproductive investment when paired with low-quality females (*negative differential allocation*). This hypothesis has been proposed for monogamous species with biparental care, and most empirical studies focus on birds. Here we used the polygamous spider *Paratrechalea ornata*, in which males offer prey wrapped in silk as nuptial gifts, to test whether males adjust their reproductive investment in gift size, pre-copulatory and copulatory courtship, and sperm transfer in response to female body condition.

**Results:**

Males exposed to females in good body condition added more flies to the gift, stimulated these females longer with abdominal touches during pre-copulatory courtship, and had longer pedipalp insertions than males exposed to females in poor body condition. Female condition affected neither silk investment in nuptial gift wrapping nor the quantity of sperm transferred by males. Finally, females in good body condition oviposited faster after copulation and laid more eggs than females in poor body condition.

**Conclusions:**

We provide experimental evidence that males of a gift-giving spider exhibit positive differential allocation in three key aspects of their reproductive investment: the size of the nutritious gift, duration of pre-copulatory courtship, and duration of pedipalp insertions, which is regarded as a form of copulatory courtship in spiders. This positive differential allocation is likely associated with the benefits of copulating with females in good body condition. These females are more fecund and oviposit faster after copulation than females in poor body condition, which under natural field conditions probably reduces the risk of multiple matings and thus the level of sperm competition faced by the males. As a final remark, our findings indicate that the hypothesis of differential allocation also applies to species with a scramble competition mating system, in which males heavily invest in nuptial gift construction, but not in parental care.

**Supplementary Information:**

The online version contains supplementary material available at 10.1186/s12862-021-01870-1.

## Background

The ability to choose the best mating partner is a critical factor for the reproductive success of an individual [1, 2]. Due to early assumptions about sex roles, mate choice has been historically studied mainly in females [3]. However, there is increasing evidence that males of many species also choose their mating partners (reviewed in [4] and [5]). Current theory predicts that male mate choice should evolve when: (1) the mate encounter rate is high, (2) there is great variation among females in traits associated with quality (e.g., fecundity), (3) mate searching effort is relatively inexpensive for males, and (4) males perform substantial reproductive investment or suffer from sperm depletion so that they are unable to mate with many available females [5, 6]. For instance, in the pipefish *Syngnathus typhle* (Syngnathidae), a species with exclusive paternal care and limited mating opportunities, males are selective when there are more females than males in the population, preferring the larger and more fecund females. However, males mate indiscriminately when there are fewer females than males in the population, probably because the mate searching costs are too high [7].

In species showing male mate choice, males can reject females of low-quality or, in a more subtle way, adjust their reproductive investment according to female quality, a behavior known as cryptic male choice [4]. When individuals increase their reproductive investment in response to high-quality mates, we call it *positive differential allocation* [8, 9]. For instance, there is empirical evidence for some species of insects, crustaceans, fish, and birds showing that males increase ejaculate volume or the quantity of sperm cells when mating with high-quality females, which may be more fecund or more ornamented [e.g., 10, 11, 12]. In contrast, when males increase their reproductive investment in response to low-quality mates, we call it *negative differential allocation* [9]. For instance, in the blue tit *Cyanistes caeruleus* (Paridae), a species with biparental care, males increase the investment in paternal care when paired with low-quality females [e.g., 13]. In this case, the higher paternal effort may be regarded as a form reproductive compensation that increases offspring production and/or survival when males are paired with females of low genetic quality, which are those with less intense ultra-violet plumage [9, 13].

Species in which males offer nuptial gifts are good models to investigate male mate choice and differential allocation for at least two main reasons. First, edible gifts with nutritive value are usually expensive and represent substantial reproductive investment for males, which may reduce their mating opportunities [e.g., 14, 15, 16, 17]. The costs associated with the production of a single gift in some insect species are so high that males take as much as five days to replenish it, and during this period they are prevented from mating [e.g., 18]. Considering that the cost of producing nuptial gifts limits the number of copulations, males should mate mainly with high-quality females if the availability of potential mating partners is high and female quality shows great variation in the population [e.g., 14, 19, 20]. Second, the nutrients of nuptial gifts may be regarded as a form of paternal effort (*sensu* [21]) if they provide direct benefits to females, increasing their fecundity [22]. Considering that paternal effort is subject to differential allocation [8, 9], males should adjust their investment in the size and/or content of the nuptial gifts in response to the quality of the mating partners [23]. In case of a positive differential allocation, males should offer larger and/or more nutritive gifts when paired with high-quality females, thus increasing their mating chances. In turn, if males exhibit negative differential allocation, they should offer larger and/or more nutritive gifts when paired with low-quality females, thus providing resources to be used in egg production. In both cases, larger and/or more nutritive gifts can also prolong copulation, which may increase the amount of sperm transferred to the female [e.g., 24].

Most empirical studies on male mate choice in gift-giving arthropods focus on orthopterans that produce endogenous gifts, known as spermatophylax, which is released together with the spermatophore (reviewed in [25]). Given that the spermatophylax is already formed when a male finds a potential mating partner [22], he is probably unable to adjust the size and/or content of the gift in response to female quality (but see [26]). However, not all endogenous gifts are pre-formed, opening the possibility that their size and/or content can be adjusted by the males in response to female quality. In fact, males of the scorpionfly *Panorpa cognata* (Panorpidae) adjust the size of the salivary mass (an endogenous gift) based on female body condition, but only when they are in poor body condition [27]. Thus, the investment in some types of endogenous gifts can be flexible, responding to both female and male condition. This finding raises the question of whether the investment on exogenous gifts, such as prey items [25], can also be adjusted adaptively by the males. This is one of the gaps we intend to fill in our study.

Several spider species construct exogenous gifts consisting of items wrapped in silk that include either nutritive prey or inedible prey leftovers [e.g., 28, 29, 30, 31]. One of these species is *Paratrechalea ornata* (Trechaleidae), in which males construct prey-gifts when they perceive chemical cues of the draglines left by conspecific females on the substrate [32]. Experimental evidence shows that prey-gift construction is costly because males in poor body condition consistently eat the prey instead of wrapping it in silk to construct a gift [33]. Despite the costs, the production of nuptial gifts is necessary for the male to be accepted by the female [34, 35]. Moreover, chemicals deposited by the male on the silk layer surrounding the gift entice the female to grab it [36], and the larger the gift, the longer copulation duration is [37]. Finally, the consumption of nutritive prey-gifts by females increases their fecundity, indicating that the offspring receives part of the nutrients contained in the gift [38]. Taken together, these findings indicate that prey-gifts in *P. ornata* entice females to copulate (i.e., mating effort) and provide food resources that enhance offspring production (i.e., paternal effort). Thus, if males can adjust the size of their nuptial prey-gifts and the quantity of silk added on them in response to female phenotypic traits, males could exhibit either positive or negative differential allocation.

Here we explored if *P. ornata* males adjust their reproductive investment in response to female quality, measured as body condition, which is known to have a marked effect on female fecundity (e.g., [39, 40]) and offspring performance in spiders (e.g., [41, 42]). We created two experimental groups of females: high-quality females (i.e., those in *good body condition*) and low-quality females (i.e., those in *poor body condition*). Then, we paired these females with males in good body condition and quantified their reproductive investment. We used three measurements of male reproductive investment: (1) the quality of the prey-gift, measured as the quantity of both prey and silk added to the gift, (2) the duration of pre-copulatory courtship, which is a crucial component of male mating effort in *P. ornata* and other gift-giving spiders [30], and (3) the copulation duration and the quantity of sperm transferred to the female, which are associated with post-copulatory processes in spiders, such as cryptic female choice (e.g., [43, 44]) and sperm competition (reviewed in [45]). If males exhibit positive differential allocation, we expect higher investment in high-quality females than low-quality females. In turn, if males exhibit negative differential allocation, we expect the opposite response, with higher investment in low-quality females than high-quality females. Finally, to evaluate the potential benefits of male differential allocation, we quantified the latency between copulation and oviposition and the number and mass of eggs laid by low- and high-quality females. We expected that high-quality females would have a shorter latency between copulation and oviposition and would lay a larger number of eggs with higher mass than low-quality females.

## Methods

### Collection and maintenance

We visited two rivers belonging to the same basin between August 22nd and 24th, 2019, in the municipality of Picada Café, state of Rio Grande do Sul, southern Brazil (29° 27′ 8.64″ S; 51° 7′ 7.42″ W and 29° 27′ 10.82″ S; 51° 2′ 37.30″ W). On the riverbanks, we collected juvenile and subadult males and females of *Paratrechalea ornata* and placed each spider in individual centrifuge tubes (50 mL) with wet cotton as a water source. Then, we transported them to our laboratory at Universidade de São Paulo (São Paulo, Brazil), where we kept the temperature around 25 ℃ and an inverted light-dark cycle of 12:12 h during the entire period of the experiment. In the laboratory, we placed the spiders individually inside larger plastic pots (200 mL) covered with a fabric mesh. While the spiders were juveniles, we fed them three times a week with one cricket nymph (*Gryllus* sp.) about 3–5 mm long.

### Conditioning period

A week after individuals molted to adulthood, we photographed each of them (n = 197) in dorsal view and measured the cephalothorax width in its wider portion using the software *ImageJ* [46]. The repeatability of the cephalothorax width measurements for both males and females was higher than 90 % (Additional file 1: Table S1). After measuring all individuals, we divided unmated females into two experimental groups: females in good body condition (i.e., *high-quality females*, hereafter ‘GOOD females’) and females in poor body condition (i.e., *low-quality females*, hereafter ‘POOR females’). We fed GOOD females and males with one cricket nymph (about 5–10 mm long) three times a week for three weeks. In contrast, we fed POOR females with a single cricket nymph (about 5–10 mm long) once a week for three weeks. Although the size of the crickets showed great variation due to weekly availability of nymphs in our stock population, the nymphs offered to POOR and GOOD females had always similar sizes in any given week. We also divided unmated males (n = 60) into two experimental groups: those exposed to GOOD females (n = 27) and those exposed to POOR females (n = 33). To avoid undesirable differences in the mean size of the individuals (females and males) between groups, we considered the cephalothorax width when we split them into the two experimental groups (Additional file 1: Table S2).

After three weeks of conditioning, we weighed males and females using a digital scale (Shimazu AUW220) to the nearest 0.0001 g. Using body weight and cephalothorax width, we performed a linear regression for females and males independently. The residuals of this linear regression are a good proxy of body condition in spiders, so that positive values indicate individuals in good body condition whereas negative values indicate individuals in poor body condition [47, 48]. In fact, GOOD females showed positive residual values that were significantly higher than the values of POOR females, which showed negative residual values. For the males, we found no difference in residual values between individuals exposed to females of each experimental group (Additional file 1: Table S2).

### Experimental setup

All individuals used in the experiment (both males and females) were 35 ± 4 (mean ± SD) days old after maturation molt. The couples were paired assortatively according to their size, so that the difference in cephalothorax width between males and females was similar for all couples in both experimental groups (Additional file 1: Table S2). The experiments were conducted during October 2019, and the trials started at 14:00 h (3 h after the beginning of the dark cycle) and finished at 23:00 h (just before the beginning of the light cycle). One to four trials were conducted each day, alternating the order of the experimental groups. The trials occurred in a circular arena with 15 cm diameter and 3 cm depth, with the floor covered with a single sheet of filter paper. The arena was divided into two halves by a removable glass barrier: the *male half* and the *female half*. During the trials, we kept the arena covered with a glass lid that allowed us to record the trials from above using a video camera (Sony HDR-CX405). To obtain more details of the behavioral interactions between males and females, we also recorded the trials laterally with another camera (Olympus Tough TG-6). Using the video recordings, we extracted all behavioral data described in the following topics. After each trial, we cleaned the entire arena with 70 % ethanol and replaced the filter paper.

### Male investment in prey-gift

Three days before the beginning of the experiment, we stopped feeding males and females of both experimental groups. Then, we placed a female in the male half of the arena for 24 h before the beginning of the trial. During this period, the female adds silk threads on the filter paper and this silk stimulates the male to construct the gift [32]. A few minutes before the beginning of the trial, we moved the female to the female half and placed the male in the male half of the arena. At this point, the male and the female could not touch each other, but they were able to see each other through the glass barrier and perceive substrate-borne vibrations that are used in pre-copulatory interactions of some spider species [49], including the gift-giving *Pisaura mirabilis* (Pisauridae) [50].

After 5 min of acclimation of the male in the arena, we placed approximately 40 (mean ± SD = 41.7 ± 4.6) fruit flies (*Drosophila melanogaster*) inside the male half. Once the male captured the first fly, we allowed him to catch other flies and add them to the gift for 1 h. After this period, we removed the remaining flies that were not added to the gift and allowed the male to add silk to the gift for another 10 min. We counted the number of flies captured by each male and recorded the time invested in adding silk to the gift. We also recorded the time spent by the female close to the glass barrier (i.e., when 100 % of her cephalothorax and abdomen were less than 3 cm away from the barrier). When females were close to the barrier, we assume that males could visually evaluate female size and/or perceive short-range vibratory signals, which are known to be a condition dependent trait in other gift-giving spiders [50]. We selected the value of 3 cm away from the barrier because it was the longest distance a female was observed moving in response to male movements on the other side of the glass barriers.

### Male investment in pre-copulatory courtship

After the end of the first phase of the trial (i.e., *Male investment in prey-gift*), we removed the glass barrier allowing physical contact between male and female. If neither the female nor the male moved during 10 min, we gently touched the female with a brush so that she approached the male and the pre-copulatory courtship initiated. We recorded the time spent by the male touching the female abdomen with his first pair of legs (‘quick touching’ *sensu* [30]), a behavior that characterizes male investment in pre-copulatory courtship in *P. ornata*. The total time spent by the male touching the female abdomen was estimated as the sum of all touching bouts during pre-copulatory courtship. We also recorded the total duration of the mating interaction, from gift acceptance by the female until the couple’s separation. Finally, as part of the male investment in prey-gift (previous topic), we recorded the time spent by the male adding more silk to the gift after he established physical contact with the female.

### Male investment in copulation duration and sperm transfer

We recorded the duration of each successful pedipalp insertion, defined as any instance in which the tip of one of the male pedipalps was in contact with the female epigyne (genital opening), and the increase in the male’s internal hydraulic pressure kept erected his leg spines for a few seconds. During the copulatory phase, a male may perform multiple insertions with both pedipalps, and we summed the time of all individual insertions to have a variable called ‘total duration of pedipalp insertions’. Although the total duration of pedipalp insertions may be positively correlated with the total quantity of sperm transferred by the males, this is not a general pattern in spiders (reviewed in [45]). In some species, for instance, the total duration of pedipalp insertions shows no correlation with the total quantity of sperm transferred by the males (e.g., [44]). Because we had no *a priori* information on whether the total duration of pedipalp insertions was correlated or not with the total quantity of sperm transferred by *P. ornata* males [51], we decided to use these two variables independently in our analyses (see below).

After copulation, we sacrificed the experimental males and photographed both their pedipalps in ventral view under a stereomicroscope. Based on these photographs, we used the software *ImageJ* [46] to measure some morphological traits that could explain the quantity of sperm stored in each pedipalp: (1) the area of the bulb, (2) the area of the median apophysis, (3) the area of the tegulum, and (4) the area of the subtegulum (Additional file 1: Fig. S1). For each trait, we estimated the repeatability of the measurements in a sample of 20 males using three measurements of each pedipalp (Additional file 1: Table S1). If a trait had repeatability higher than 90 % in this sample, we measured this trait only once in all other males; otherwise, we measured the trait three times and then calculated the mean of these values.

After photographing the pedipalps, we conserved them individually under − 80 ℃. We quantified the sperm stored in each pedipalp following the procedure proposed by Bukowski & Christenson [52]. In summary, we first placed the dissected bulb of each pedipalp into a centrifuge tube with 100 µL of a solution containing 1 ml of 0.9 % saline solution and 10 µL of 10 % triton-x detergent. Then, we grinded them with metal forceps for approximately 90 s. Next, we did three cycles of 90 s in a vortex and 25 min of centrifugation at 1000*g* and 25 ℃. Finally, we placed two samples of 10 µL in a Neubauer improved double-chamber hemocytometer. For each of the two samples, we used a microscope at 200x to count the sperm cells in the four corners of the chamber (16 squares in each corner) and summed them to obtain the number of sperm cells in the sample. Finally, we used the mean cell count of the two samples to estimate the number of sperm cells in 1 mL using the equation: (1 mL × number of sperm cells counted)/0.004 mL.

We repeated all the procedures described above with a sample of 20 unmated males that were not included in the experiment. The data obtained for these males provided us an estimation of the quantity of sperm present in each pedipalp *before* copulation. Thus, considering the size of some pedipalpal and body traits, we could infer the total quantity of sperm transferred by the males during the experiment (see details of this procedure in the topic *Statistical analyses* below).

### Potential benefits of male mate choice

After copulation, we fed both POOR and GOOD females with one cricket nymph (about 5–10 mm long, depending on their weekly availability in our stock population) three times a week until they laid their eggs. After oviposition, we waited 15 days to allow the eggs to develop so that we could distinguish fertilized from unfertilized eggs. Then, we preserved the females and their egg-sacs in 70 % ethanol. Under a stereomicroscope, we counted the total number of eggs inside each egg-sac. To weigh the eggs, we placed them on filter paper for 24 h to dry the ethanol. Finally, we weighed separately the fertilized and unfertilized eggs using a digital scale (Shimazu AUW220) to the nearest 0.0001 g. Unfertilized eggs have a diameter similar to fertilized eggs, but their content includes only a homogenous yellow yolk, and not a pre-formed spiderling, which can be easily seen through the chorion of the egg. Dividing the total mass of fertilized eggs by the total number of fertilized eggs, we obtained the mean mass of fertilized eggs laid by each female.

### Statistical analyses

#### Male investment in prey-gift

To test whether males adjust the number of flies added to the prey-gift in response to female body condition, we used a generalized linear model (GLM) with quasibinomial distribution of errors and logit as link function. The response variable was the proportion of captured flies in relation to the total number of flies offered to each male. Given that the number of flies offered to all males was approximately 40, the higher the proportion of flies captured and added to the gift, the greater its size. As predictor variables, we used the experimental groups (POOR × GOOD), the time the female spent close to the glass barrier (which represents the visual and/or short-range vibratory stimulus received by the male), and the interaction between these two variables.

To test whether males adjust the time adding silk to the gift (i.e., another measurement of male investment in the prey-gift) in response to female body condition, we performed three analyses. First, we used a GLM with gamma distribution of errors and identity as link function to test whether the time adding silk to the prey-gift *before* any physical interaction with the female was affected by the experimental groups, the time spent by the female close to the glass barrier, and the interaction between these two variables. Second, we used a GLM with binomial distribution of errors and logit as link function to estimate the probability the male adding more silk to the prey-gift *after* physical contact with the female. The predictor variables were the experimental groups, the time spent adding silk to the gift before physical contact with the female, and the interaction between these two variables. Finally, we used a GLM with gamma distribution of errors and identity as link function to test whether the *total* time spent adding silk to the gift (i.e., *before* and *after* physical contact with a female) was affected by the experimental groups. As the gamma distribution does not accept zero values, we added an infimum value (1^− 16^) to the observed values of the response variable. As the number of flies in the prey-gift was not correlated with the silk response variables of the three models (Additional file 1: Table S3), we did not include it as a predictor variable in the models.

#### Male investment in pre-copulatory courtship

To test whether males adjust the duration of pre-copulatory courtship in response to female body condition, we used a GLM with gamma distribution of errors and identity as link function. Our proxy for the duration of pre-copulatory courtship was the total time invested by a male touching a female’s abdomen (hereafter ‘abdominal touches’). As predictor variables, we used the experimental groups, the duration of the mating interaction (starting with gift acceptance and finishing with the couple’s separation), and the interaction between these two variables. Finally, as an exploratory analysis, we investigated whether male investment in pre-copulatory courtship is affected by differences between experimental groups in female receptivity. We used a GLM with negative binomial distribution of errors and log as link function. The experimental groups were the predictor variable, and the response variable was the latency between the first abdominal touch performed by the male and the abdominal twist performed by the female that exposes her genital opening to allow copulation (hereafter ‘latency to pedipalp insertion’).

#### Male investment in copulation duration and sperm transfer

To test whether males adjust the duration of pedipalp insertions in response to female body condition, we used a GLM with gamma distribution of errors and identity as link function. The total duration of pedipalp insertions was the response variable, and the predictor variables were the experimental groups, the duration of the mating interaction (starting with gift acceptance and finishing with the couple’s separation), and the interaction between these two variables.

To determine the total quantity of sperm transferred to the females, we first estimated the quantity of sperm stored in each pedipalp *before* copulation. To do that, we used only the unmated males to find the best combination of morphological traits to describe the quantity of sperm stored in each pedipalp. We divided our morphological traits into body-related traits, including cephalothorax width and body condition, and pedipalp-related traits, including the areas of the bulb, median apophysis, tegulum, and subtegulum. We ran models with different distributions of errors (Gaussian, Poisson, and negative binomial), always using the quantity of sperm (number of sperm cells/mL) stored in each pedipalp as the response variable. As predictor variables, we included combinations of three or fewer morphological traits (body- and/or pedipalp-related) with additive and interactive effects between them (for further details see *Methods of sperm quantification* in Additional file 1). In total, we had 174 concurrent models and used the Akaike Information Criterion corrected for small samples (AIC_c_) to select the best model, i.e., the one that includes the morphological trait(s) that better predict the quantity of sperm stored in each pedipalp (Additional file 1: Table S4).

As an alternative approach, we performed a Principal Components Analyses (PCA) to reduce the dimensionality of the morphological traits. Using combinations of one to three Principal Components (PCs), we ran models with different distributions of errors and including both additive and interactive effects between predictor variables (Additional file 1: Tables S5–S7). Given that the best model using PCs explained less variability of the data (17.7%) than the best model using morphological traits (20%), the procedure we used to estimate the quantity of sperm transferred by the males is based on the data obtained with morphological traits.

According to the analysis performed with morphological traits, we found two models with ΔAIC_c_ < 2: (1) *sperm stored* = *cephalothorax width*, and (2) *sperm stored* = *cephalothorax width* + *subtegulum area* (Additional file 1: Table S4). For the sake of simplicity, we used model (1), which has only one predictor variable, also included in model (2). Based on the equation obtained using model (1), we estimated the quantity of sperm stored in each pedipalp of the experimental males before copulation. By subtracting the quantity of sperm in the pedipalp *after* copulation (i.e., mated males) from the estimation of sperm stored in the pedipalp *before* copulation (obtained with unmated males), we had an estimation of the quantity of sperm transferred to the female by each pedipalp. In only one case, the estimation of the total sperm transferred had a slightly negative value, and we approximated it to zero, considering that there was no sperm transference. For each male, we summed the quantity of sperm transferred by each pedipalp and used this value as our response variable.

To test whether males adjust the quantity of sperm transferred in response to female quality, we used a generalized least square (GLS) model. The predictor variables were the experimental groups, the total duration of pedipalp insertion, and the interaction between these two variables. As an exploratory analysis, we also tested whether the quantity of sperm transferred by the males was related to the number of fertilized eggs laid by the females. To do so, we used a GLM with gamma distribution of errors and identity as link function.

#### Potential benefits of male mate choice

To determine whether GOOD females provide greater benefits to males than POOR females, we used three proxies: (1) the latency between copulation and oviposition, (2) the total number of eggs laid by the female, and (3) the mean mass of fertilized eggs. The rationale for the first proxy is that the lower the latency between copulation and oviposition, the lower the risk of the female copulating with other males, and thus the lower the sperm competition risk and/or intensity faced by the male. We lost information about some POOR (n = 8) and GOOD females (n = 14) that either died before oviposition or ate their egg-sac immediately after oviposition. Therefore, our sample size was 25 POOR females and 18 GOOD females for the test on the latency between copulation and oviposition, and 25 POOR females and 13 GOOD females for the tests on the total number of eggs and the mean mass of fertilized eggs.

To test whether GOOD females lay eggs faster than POOR females, we ran models with different distributions of errors (Gaussian, Poisson, and gamma), always using the number of days between copulation and oviposition as the response variable. We ran five models to each distribution of errors with different combinations of predictor variables: (1) experimental groups, (2) number of flies added to the gift, (3) additive effect of experimental groups and number of flies added to the gift, (4) interactive effect of experimental groups and number of flies added to the gift, and (5) null model. We used the Akaike Information Criterion corrected for small samples (AIC_c_) to select the best fitted model. We used the same approach to test whether GOOD females lay more eggs and heavier fertilized eggs than POOR females. In these two analyses, we used as response variables the total number of eggs laid by the females and the mean mass of fertilized eggs (mg), respectively. As the mean mass of fertilized eggs is a continuous variable, we only used Gaussian and gamma distributions of errors in the model selection.

#### Software and packages

We ran all analyses in the software R 4.0.3 [53], using the packages *stats* [53] and *MASS* [54] to perform the GLMs, the package *bbmle* [55] to perform the model selection, and the package *nlme* [56] to perform the GLS.

## Results

### Male investment in prey-gift

**Fig. 1:**
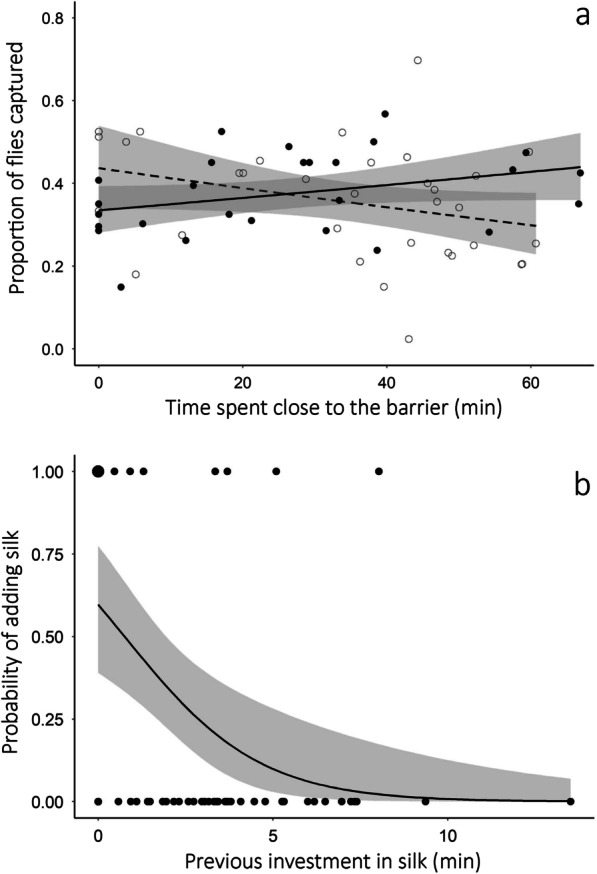
Investment in **a** flies and **b** silk added to the nuptial gift by males of the spider *Paratrechalea ornata*. **a** Proportion of flies captured by males exposed to females in POOR condition (white dots) and GOOD condition (black dots). Given that we offered approximately 40 flies for each male, the higher the proportion of flies added to gift, the larger it is. The time spent by the females close to the glass barrier in the experimental arena was included a continuous predictor variable because it represents the visual and/or short-range, substrate-borne vibratory stimulus received by the males during the pre-copulatory phase. Lines indicate the tendency predicted for males exposed to each experimental group: dashed = females in POOR condition; solid = females in GOOD condition. **b** Probability of a male adding more silk to the gift *after* physically interacting with the female in response to his previous investment in silk (i.e., the time spent adding silk to the gift *before* physically interacting with the female). Circle sizes are proportional to the number of superimposed data points. In both graphics the shaded area indicates the 95 % confidence interval

Males captured 1–27 flies to construct the gift. There was an interaction between the experimental groups and the time females spent close to the glass barrier (Table 1). When males were exposed to POOR females, the proportion of flies added to gift decreased with the time females spent close to the glass barrier. In turn, when males were exposed to GOOD females, the proportion of flies added to the gift increased with time females spent close to the glass barrier (Fig. 1a).

**Table 1:** Summary of the statistical models used to explain male investment in flies and silk added to the nuptial gift

Predictors	Estimate	SE	t- or z-value	p-value
* Proportion of flies added to the gift *				
Intercept (POOR females)	− 0.28 (0.43)	0.18	− 1.54	0.130
GOOD females	− 0.41 (0.40)	0.24	− 1.71	0.093
Time near the barrier	− 0.01 (0.50)	0.005	− 2.080	**0.042**
GOOD females × Time near the barrier	0.02 (0.50)	0.007	2.498	**0.016**
* Time adding silk before physical interaction *				
Intercept (POOR females)	3.05	1.06	2.878	0.006
GOOD females	0.01	0.02	0.236	0.814
Time near the barrier	− 1.59	1.18	− 1.35	0.182
GOOD females × Time near the barrier	0.03	0.03	0.897	0.373
* Probability of adding more silk after physical interaction *				
Intercept (POOR females)	0.57 (0.64)	0.58	0.99	0.051
GOOD females	− 0.38 (0.41)	0.87	− 0.435	0.658
Time previously invested in silk	− 0.52 (0.37)	0.22	− 2.411	**0.016**
GOOD females × Previous invest in silk	0.01 (0.50)	0.35	0.038	0.970
* Total time adding silk *				
Intercept (POOR females)	3.79	0.50	7.597	< 0.001
GOOD females	− 0.77	0.67	− 1.149	0.255

For the models of *Proportion of flies added to the gift* and *Probability of adding more silk before physical interaction*, we report the estimate in logit units and present the original units in parentheses. Moreover, the model of *Probability of adding more silk before physical interaction* uses a z-value instead of a t-value as in the other models. Significant results are highlighted in bold. SE = standard error

Fourteen males (23 % of the total) did not add silk to the gift *before* physically interacting with the female, but 10 of these males added silk to the gift *after* physically interacting with the female. Males exposed to POOR females invested a total of 3.8 ± 3.1 min (mean ± SD) adding silk to the gift, whereas males exposed to GOOD females invested a total of 3.0 ± 2.1 min. There was no difference in the time males invested adding silk to the gift (both *before* the physical interaction and *total*) when they were exposed to POOR or GOOD females (Table 1). However, the probability of the male adding more silk *after* physically interacting with the females increased when the previous investment in silk was low (Fig. 1b; Table 1).

### Male investment in pre-copulatory courtship

**Fig. 2:**
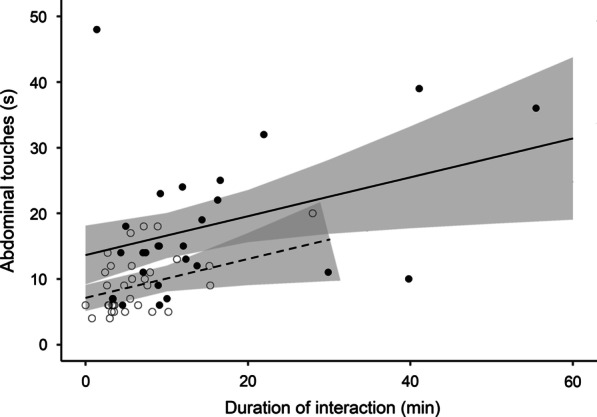
Time spent by males of the spider *Paratrechalea ornata* touching the female abdomen according to the total duration of the mating interaction. White dots represent males exposed to females in POOR condition and black dots represent males exposed to females in GOOD condition. Lines indicate the tendency predicted for males exposed to each experimental group: dashed = females in POOR condition; solid = females in GOOD condition. The shaded area indicates the 95 % confidence interval

**Fig. 3:**
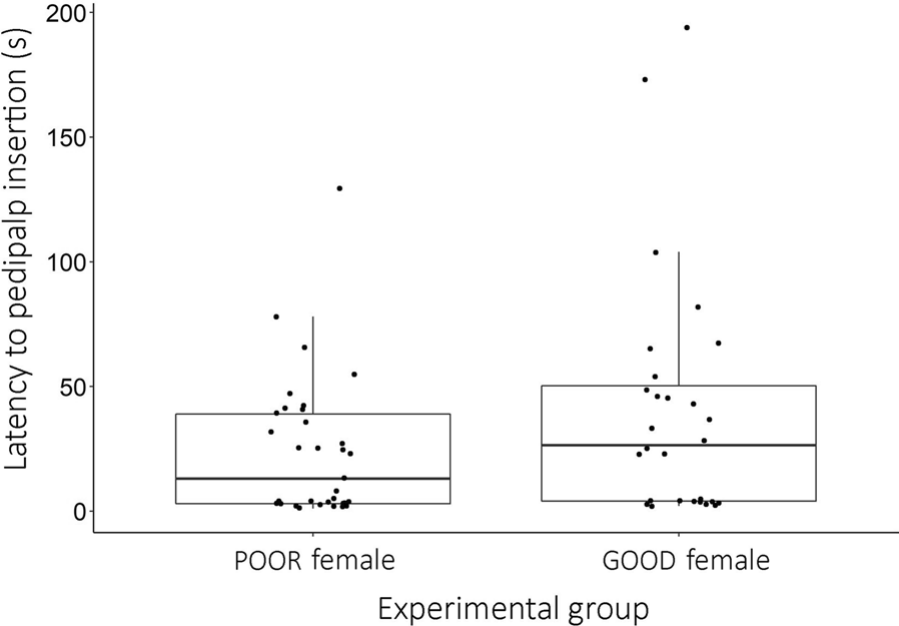
Latency to pedipalp insertion in the spider *Paratrechalea ornata* according to female body condition. The latency is the interval between the first abdominal touch performed by the male and the abdominal twist performed by the female that exposes her genital opening to allow copulation. The boxes contain 50 % of the data and the line inside each box represents the median

All males stimulated the females with abdominal touches (n = 60). Males exposed to POOR females spent 9.2 ± 4.4 s (mean ± SD) stimulating the abdomen of the partner, whereas males exposed to GOOD females spent 18.2 ± 10.7 s. Males exposed to GOOD females spent more time stimulating females with abdominal touches than males exposed to POOR females (Fig. 2; Table 2). Moreover, the longer the mating interaction, the more abdominal touches the males performed (Fig. 2; Table 2). There was no difference between experimental groups in the latency to pedipalp insertion, i.e., between the first abdominal touch performed by the male and the abdominal twist performed by the female that exposes her genital opening to allow copulation (Fig. 3; Table 2).

**Table 2:** Summary of the statistical models used to explain pre-copulatory (*Abdominal touches* and *Latency to pedipalp insertion*) and copulatory (*Total duration of pedipalp insertions* and *Total sperm transferred*) investment by males exposed to females in POOR and GOOD condition

Predictors	Estimate	SE	t-value	p-value
* Abdominal touches *				
Intercept (POOR females)	6.17	1.26	4.905	< 0.001
GOOD females	8.43	2.79	3.022	**0.004**
Duration of the mating interaction	0.49	0.22	2.215	**0.031**
GOOD females × Duration of the mating interaction	− 0.27	0.26	− 1.047	0.300
* Latency to pedipalp insertion *				
Intercept (POOR females)	3.18 (24.15)	0.20	15.715	< 0.001
GOOD females	0.51 (1.67)	0.30	1.719	0.085
* Total time of pedipalp insertion *				
Intercept (POOR females)	0.65	0.25	2.63	< 0.001
GOOD females	2.04	0.46	4.41	**< 0.001**
Duration of interaction	0.16	0.05	2.98	**0.004**
GOOD females × Duration of interaction	− 0.19	0.05	− 3.51	**< 0.001**
* Total sperm transferred *				
Intercept (POOR females)	50,846.11	7262.34	7.00	< 0.001
GOOD females	2889.66	8627.38	0.33	0.739
Total time of pedipalp insertion	4881.56	3669.48	1.33	0.189
GOOD females × Total time of pedipalp insertion	− 2599.45	4033.99	− 0.64	0.522

In the model of *Abdominal touches*, the total duration of the mating interaction (starting with gift acceptance and finishing with the couple’s separation) was included as continuous predictor. For the model of *Latency to pedipalp insertion*, we report the estimate in log units and the original units are presented in parentheses. Significant results are highlighted in bold. SE = standard error

### Male investment in copulation duration and sperm transfer

**Fig. 4:**
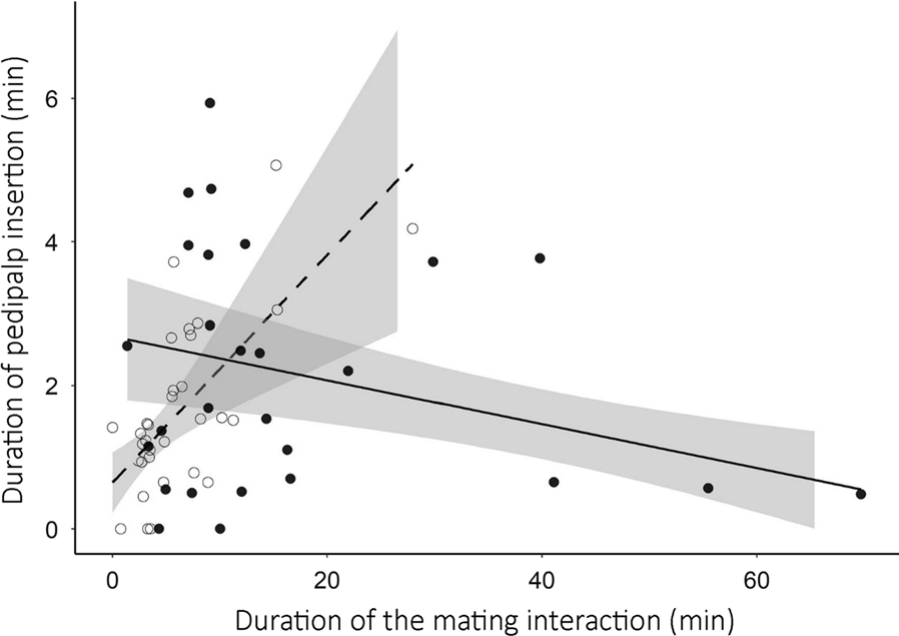
Time spent by males of the spider *Paratrechalea ornata* in pedipalp insertions into the female epigyne (genital opening) according to the total duration of the mating interaction. White dots represent males exposed to females in POOR condition and black dots represent males exposed to females in GOOD condition. Lines indicate the tendency predicted for males exposed to each experimental group: dashed = females in POOR condition; solid = females in GOOD condition. The shaded area indicates the 95 % confidence interval

Males performed from zero to five pedipalp insertions during the copulatory phase. They usually alternated pedipalps between insertions, and each insertion lasted from 0.33 to 12.22 min. The total duration of pedipalp insertions was longer for males exposed to GOOD females than males exposed to POOR females (Fig. 4; Table 2). Moreover, the total duration of pedipalp insertions decreased in longer mating interactions, but only for males exposed to GOOD females (Fig. 4). For males exposed to POOR females, the total duration of pedipalp insertion increased in longer mating interactions (Fig. 4). Regarding the total quantity of sperm transferred to females, there was no difference between males exposed to POOR and GOOD females (Table 2). Moreover, there was no correlation between the total duration of pedipalp insertions and the quantity of sperm transferred to the females (Table 2). Finally, there was also no correlation between the quantity of sperm transferred to the females and the number of fertilized eggs laid by these females (t = 0.90, df = 32, p = 0.375).

### Potential benefits of male mate choice

**Fig. 5:**
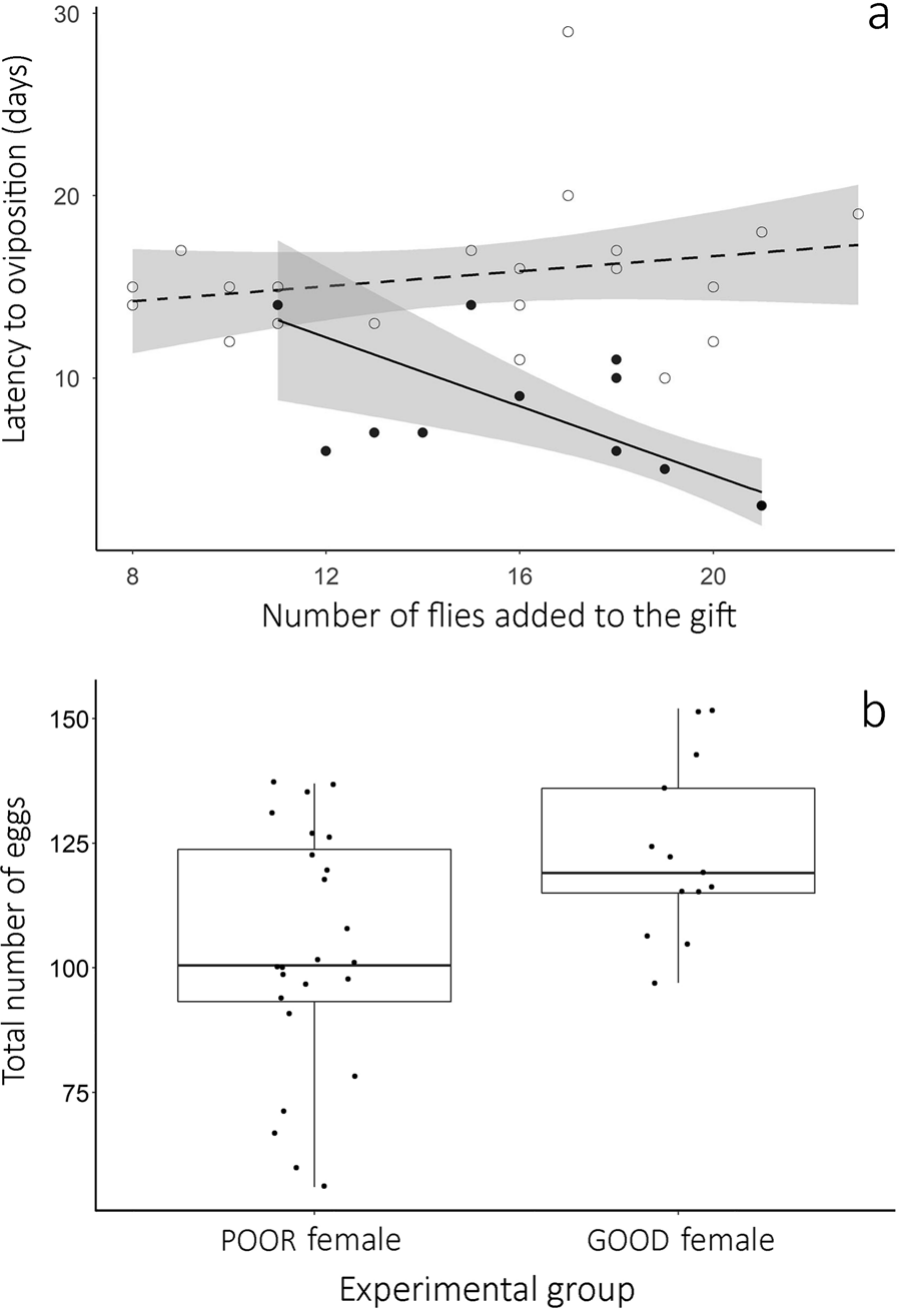
Potential benefits of differential allocation in reproductive investment when males of the spider *Paratrechalea ornata* are exposed to females in POOR and GOOD condition. **a** Latency between copulation and oviposition according to female condition and the number of flies added to the gift. White dots represent males exposed to females in POOR condition and black dots represent males exposed to females in GOOD condition. Lines indicate the tendency predicted for males exposed to each experimental group: dashed = females in POOR condition; solid = females in GOOD condition. The shaded area indicates the 95 % confidence interval. **b** Boxplot of the total number of eggs laid by females according to their condition. The boxes contain 50 % of the data and the line inside each box represents the median

We found two models with ΔAIC_c_ < 2 to explain the latency between copulation and oviposition. Both models include an interaction between the experimental groups and the number of flies added to the gift, differing only in the type of distribution of errors (Additional file 1: Table S8). Because the Akaike weight of the best fitted model with Poisson distribution of errors is almost two times higher than that of the model with gamma distribution of errors, we used the former to estimate the parameters presented in Table 3. In general terms, the latency between copulation and oviposition was shorter for GOOD females when the males added a greater number of flies to the gift (Fig. 5a). For POOR females, the number of flies added to the gift did not affect the latency between copulation and oviposition (Fig. 5a).

**Table 3:** Summary of the statistical models used to evaluate the potential benefits provided by females in POOR and GOOD condition

Predictors	Estimate	SE	z- or t-value	p-value
* Latency to oviposition *				
Intercept (POOR females)	2.55 (12.81)	0.19	13.122	< 0.001
GOOD females	0.74 (2.09)	0.54	1.357	0.175
Number of flies added	0.01 (1.01)	0.01	1.105	0.269
GOOD females x Number of flies added	− 0.09 (0.91)	0.03	− 2.629	**0.008**
* Total number of eggs *				
Model (1)				
Intercept (POOR females)	127.77	13.53	9.296	< 0.001
GOOD females	23.80	7.72	3.084	**0.004**
Number of flies added	− 1.54	0.83	− 1.854	0.073
Model (2)				
Intercept (POOR females)	102.18	4.78	21.385	< 0.001
GOOD females	20.97	7.84	2.675	**0.011**
* Mean mass of fertilized eggs *				
Intercept	0.12	0.01	11.27	< 0.001

For the model of *Latency to oviposition*, we report the estimate in log units and present the original units in parentheses. Moreover, the model of *Latency to oviposition* uses a z-value instead of a t-value as in the other models. For the model of *Total number of eggs*, we present the results of the two best fitted models (ΔAIC_c_ < 2): model (1) includes the additive effect of the experimental groups and number of flies added to the gift, and model (2) includes only the effect of the experimental groups. Significant results are highlighted in bold. SE = standard error

We also obtained two models with ΔAIC_c_ < 2 to explain the number of eggs laid by the females (both with Gaussian distribution of errors): model (1) includes the additive effect of the experimental groups and number of flies added to the gift, and model (2) includes only the experimental groups (Additional file 1: Table S8). Although the Akaike weight of the model (1) is two times higher than that of model (2), the explanatory power of the number of flies is low and this variable is not significant in model (1) (Table 3). Thus, based on model (2), which includes only the effect of experimental groups, the number of eggs laid was higher for GOOD females (mean ± SD = 123 ± 18 eggs) compared with POOR females (103 ± 24 eggs) (Fig. 5b).

One of 13 GOOD females and five of 25 POOR females laid only unfertilized eggs. The number of fertilized eggs laid by females of both experimental groups was positively correlated with the total number of eggs laid (r = 0.65, t = 5.076, df = 35, p < 0.001). Finally, the best fitted model to explain the mean mass of fertilized eggs was the null model (Additional file 1: Table S8). The mean mass (± SD) of fertilized eggs in both experimental groups was 0.122 ± 0.063 mg.

## Discussion

Here, we provide experimental evidence that males of the gift-giving spider *Paratrechalea ornata* adjust their reproductive investment in response to females’ body condition, which was our proxy of quality. Our findings show that, during gift construction, males exposed to high-quality females (i.e., those in good body condition) capture more flies than males exposed to low-quality females (i.e., those in poor body condition). However, silk investment in the nuptial gift does not differ between males exposed to low- and high-quality females. During pre-copulatory courtship, males exposed to high-quality females stimulate longer their partners with abdominal touches compared with males exposed to low-quality females. In the copulatory phase, males exposed to high-quality females perform longer pedipalp insertions than males exposed to low-quality females, but the quantity of sperm transferred does not vary according to female quality. Finally, high-quality females have shorter latency between copulation and oviposition and lay more eggs than low-quality females.

Our results suggest that the adjustment in gift size is mediated by visual and/or short-range vibrational stimuli acquired by the male in the pre-contact phase of the mating interaction. When females are close to the glass barrier of the experimental arena, males increase the number of flies added to the gift in response to high-quality partners, while decrease the number of flies added to the gift in response to low-quality partners (Fig. 1a). The use of visual cues to access the size and body condition of potential partners has already been reported for visually oriented spiders. In wolf spiders (e.g., [57, 58]), for instance, females select males in good body condition using both visual and tactile cues. Moreover, in the gift-giving spider *Pisaura mirabilis*, females distinguish males in poor and good body condition using short-range vibrational signals emitted during pre-copulatory and copulatory courtship [50]. In the case of *P. ornata*, males may use visual cues, such as the volume of the female’s abdomen, and/or short-range vibrational signals, such as tremulation of legs or abdomen, to distinguish mating partners in poor and good body condition. Although the presence of female silk is important to trigger gift construction by *P. ornata* males [32], chemical cues alone are probably ineffective in inducing adjustments in gift size because when females are far from the glass barrier, the number of flies added to the gift is similar between males exposed to low- and high-quality females (Fig. 1a). Despite extensive empirical evidence showing that males use chemical cues in female silk to access information about her mating status (e.g., [59, 60, 61, 62]), male’s ability to access female body condition using silk cues seems to be relatively rarer in spiders (e.g., [63]).

Gift-giving is regarded as a form of mating effort when it increases male’s mating probability [22]. In *P. ornata*, the silk layer surrounding the gift contains chemicals deposited by the male that entice the female to accept the prey-gift [36]. However, our results show that males do not adjust the quantity of silk deposited on the prey-gift in response to female quality. Considering that silk production in spiders is costly [64], males may invest the minimum amount of silk to properly wrap the prey-gift and adjust the quality of the prey-gift by changing the number of prey added to it. In fact, we found that males construct larger gifts when exposed to high-quality females and smaller gifts when exposed to low-quality females (Fig. 1a). An experimental study with the scorpionfly *Panorpa cognata* has shown that males adjust the size of their *endogenous* gift (i.e., salivary mass) in response to female quality [27]. Our study is perhaps the first demonstration that males can also adjust the size of an *exogenous* gift (i.e., prey wrapped in silk) in response to female quality. Our findings expand previous studies on modulation of gift size in arthropods because we show that high-quality females indeed offer more fitness benefits to males than low-quality females. By increasing their mating effort in response to high-quality females, males have access to more eggs (Fig. 5b). Moreover, males probably face lower levels of sperm competition because the more flies they add to the gift offered to high-quality females, the lower the latency to oviposition (Fig. 5a). For the largest gifts recorded in our experiment, the latency to oviposition for high-quality females is slightly shorter than the mean remating interval (5.5 days) previously reported for well-fed females of *P. ornata* [65]. In turn, low-quality females are non-responsive to the number of flies added to the gift and their latency to oviposition is consistently longer than the mean remating interval (Fig. 5a).

Theoretical and empirical studies on differential allocation indicate that individuals can either increase or decrease their parental effort in response to the quality of their mating partners [9, 13, 66]. Previous studies with *P. ornata* show that the nuptial gift also functions as paternal effort because well-fed females that receive a nutritious gift lay more eggs than well-fed females that receive a worthless gift [38]. Thus, males could construct larger gifts when exposed to low-quality females to increase the fecundity of their partners and gain more fitness benefits. However, our results show that males construct smaller gifts when exposed to low-quality females (Fig. 1a). We suggest that these females use most of the gift’s nutrients for self-maintenance rather than egg production. In support of this suggestion, starved males of *P. ornata* prioritize self-maintenance and feed on the prey instead of constructing a nuptial gift when exposed to cues of an unmated female [33]. Our results also show that males exposed to high-quality females construct large gifts (Fig. 1a). However, the possibility that these females capitalize the nutrients of the gift for egg production is unlikely because the interval between copulation and oviposition is too short (Fig. 5a). In fact, we found that the number of flies added to the gift has no relevant effect on the number of eggs laid by the females, even when they were in good body condition. Although the gift’s nutrients could also be used to increase egg size, we found no difference in the mean mass of the fertilized eggs laid by low- and high-quality females. Thus, we argue that the adjustment in gift size reported here is more related to its function as *mating* effort than *parental* effort.

The modulation of males’ behavior in response to female quality extends to the pre-copulatory courtship, in which males exposed to high-quality females invest more in abdominal touches compared with low-quality females. Pre-copulatory courtship is an essential source of information for females to evaluate potential mates and a key factor influencing male mating success in arthropods (examples in [67]). In *P. ornata*, the abdominal touches performed by males during pre-copulatory courtship are followed by a female abdominal twist that exposes her genital opening to allow copulation [30]. In the absence of abdominal touches, copulation does not happen, suggesting that male tactile stimulation is crucial for male mating success. Males spend more time stimulating high-quality females because these females may be choosier than low-quality females. For instance, in wolf spiders of the genus *Schizocosa* and in the beetle *Callosobruchus maculatus*, females in poor body condition mate with males no matter their quality, but females in good body condition only mate with high-quality males [68, 69]. However, we found no effect of female condition in the latency to accept the first pedipalp insertion, suggesting that high-quality females of *P. ornata* are not choosier than low-quality females (Fig. 3). Thus, we interpret that the higher investment in pre-copulatory courtship is mainly related to the greater fitness benefits males may acquire by stimulating more fecund females (Fig. 5b). In the cricket *Gryllus bimaculatus*, for instance, males invest more time courting larger and probably more fecund females [70]. In *P. ornata* males, specifically, the longer pre-courtship of high-quality females compared with low-quality females can increase their chances of fertilizing a greater number of eggs, a post-copulatory process not investigated here.

Males of *P. ornata* also adjust the duration of pedipalp insertions in response to female quality, with males exposed to high-quality females showing longer insertions than males exposed to low-quality females. However, there is no difference in sperm quantity transferred to low- and high-quality females. Moreover, the duration of pedipalp insertions is not correlated with the sperm quantity transferred to the females, suggesting that the biological meaning of these two variables is not the same. In fact, a recent experimental study with the spider *Holocnemus pluchei* (Pholcidae) has shown that males with prolonged copulation have more stored sperm in the female reproductive tract, even though males with shorter copulation transfer the same quantity of sperm [44]. The authors suggest that prolonged copulation is related to the stimulation of the female reproductive tract by the male (see also [71] and [72] for additional examples with damselflies and soldier flies, respectively). This stimulation during copulation could be under cryptic female choice, so that males that provide longer stimulation are favored by females and thus increase their fertilization success [73]. If the quantity of sperm stored by *P. ornata* females is also cryptically selected based on male stimulation during copulation, sexual selection should favor males that increase the investment in copulation duration when exposed to more fecund females (i.e., females in good body condition), as we found here. Why males do not adjust the quantity of sperm transferred to the females is an open question that deserves further investigation. An interesting possibility is that females have control over the fate of the sperm inside their reproductive tract (see discussion in [51]). We argue that the lack of correlation between the quantity of sperm transferred by the males and the number of fertilized eggs laid by the females supports this suggestion. Thus, the best male strategy to increase fertilization success may be to invest in copulatory stimulation rather than in the quantity of sperm transferred to the mating partner [51].

## Conclusions

There is growing evidence that males can select their mating partners, but the subject of differential allocation in males remains poorly explored, especially in species with nuptial gifts (but see [26, 27]). Here we provide experimental evidence that males of the gift-giving spider *P. ornata* exhibit positive differential allocation in three key aspects of their mating effort: nuptial gift size, duration of pre-copulatory courtship, and duration of pedipalp insertions. This positive differential allocation is likely associated with the benefits of copulating with high-quality females, which lay approximately 20 % more eggs than low-quality females and produce a larger number of fertilized eggs. Moreover, high-quality females probably represent a lower level of sperm competition because they (1) oviposit faster after copulation and (2) may be less prone to remating than low-quality females, as reported for other gift-giving spiders [74, 75]. Both the benefits in terms of more eggs that can be fertilized, and the decreased level of sperm competition may favor the evolution of positive differential allocation by the males. As a final remark, our findings reinforce the notion that the hypothesis of differential allocation, which has been originally proposed to species with monogamous mating systems and biparental care [76], also applies to species with a scramble competition mating system in which males invest in nuptial gift construction, but not in parental care.

## Supplementary Information

**Additional file 1.** Supplementary information.

## Data Availability

The data used in this study are available at Mendeley Repository (10.17632/d28nn8gvyr.1).
