# Supplementary material for: Differential allocation in a gift-giving spider: males adjust their reproductive investment in response to female condition
Source: BMC Ecol Evol. 2021 Jul 8;21:140. doi: 10.1186/s12862-021-01870-1 (PMC8268551; doi:10.1186/s12862-021-01870-1)
Supplement: Supplementary file 1 — Additional file 1. Supplementary information. [file 12862_2021_1870_MOESM1_ESM.docx]

**Supplementary Material**

**Table S1**. Repeatability of the measurements of cephalothorax width of males and females of the spider *Paratrechalea ornata*. The table also includes measurements of the areas of the bulb, median apophysis, tegulum, and subtegulum of males’ pedipalps. We followed Lessells & Boag (1987) to calculate the repeatability using three measurements of each structure in a sample of 20 individuals.

| **Structure** | **Repeatability** |
| --- | --- |
| Female cephalothorax width | 0.984 |
| Male cephalothorax width | 0.964 |
| Area of the bulb | 0.962 |
| Area of the median apophysis | 0.979 |
| Area of the tegulum | 0.601 |
| Area of the subtegulum | 0.498 |

**Table S2.** Tests of some key assumptions of the experimental protocol. Values are presented as mean ± standard deviation.

| **Assumption** | **Experimental group** | | **Statistics** |
| --- | --- | --- | --- |
|  | **POOR** | **GOOD** |  |
| Difference in female body condition after conditioning | -0.020 ± 0.009 | 0.024 ± 0.014 | t = 14.498, df = 58, p < 0.001 |
| No difference in male body condition | 0.0014 ± 0.0082 | -0.0016 ± 0.0053 | t = -1.685, df = 58, p = 0.097 |
| No difference in female size | 3.924 ± 0.271 | 3.958 ± 0.231 | t = 0.515, df = 58, p = 0.609 |
| No difference in male size | 3.720 ± 0.205 | 3.768 ± 0.176 | t = 0.971, df = 58, p = 0.336 |
| No difference in the difference between female and male size | -0.0008 ± 0.215 | 0.0009 ± 0.225 | t = 0.030, df = 58, p = 0.976 |


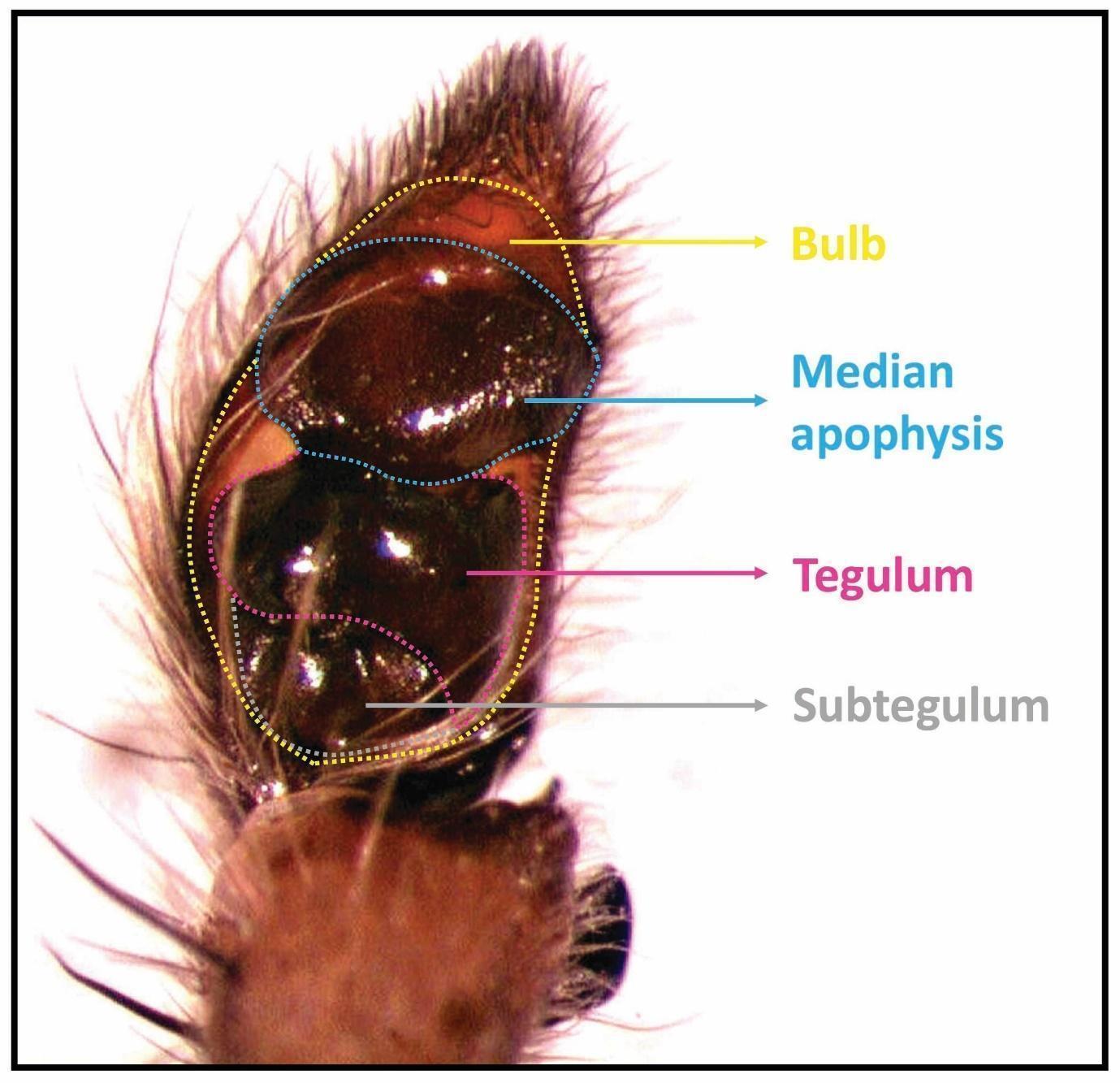


**Figure S1.** Pedipalp of a *Paratrechalea ornata* male showing the structures measured to estimate the sperm stored *before* copulation and then the quantity of sperm transferred to the female *after* copulation.

**Table S3.** Correlation between the quantity of flies added to the gift and three different variables of investment in silk added to the gift by males of the spider *Paratrechalea ornata*. For each variable we show the value of the Pearson’s correlation (*r*) and its t-value, the degree of freedom (df), and the p-value.

| **Variable** | ***r*** | **t-value** | **df** | **p-value** |
| --- | --- | --- | --- | --- |
| Silk before (min) | 0.056 | 0.426 | 58 | 0.672 |
| Silk after (min) | 0.028 | 0.217 | 58 | 0.829 |
| Total silk (min) | 0.069 | 0.524 | 58 | 0.602 |

**Methods of sperm quantification**

In our first approach, we conducted a model selection using morphological variables to predict the sperm stored in the pedipalps before copulation. The predictor variables included body traits, such as the cephalothorax width (Cephalothorax) and the residuals of a linear regression between cephalothorax width and body weight (Condition), and pedipalpal traits, such as the median apophysis area (Apophysis), tegulum area (Tegulum), subtegulum area (Subtegulum), and bulb area (Bulb). The concurrent models included all possible combinations of three or less predictor traits, with additive and interactive effects between them. To keep our capacity to detect reasonable-size effects with acceptable power, we did not include models with a combination of more predictor variables (following Harrel, 2015). For each combination we constructed models using Gaussian, Poisson, and negative binomial (NB) distribution of errors. To select the best combination of predictors to explain the quantity of sperm stored in the pedipalps, we used the Akaike Information Criteria corrected for small samples (AIC_c_).

The results of the model selection are presented in Table S4. The two best models (i.e., those with ΔAIC_c_ < 2) contained “Cephalothorax” and “Cephalothorax + Subtegulum” as predictor variables. Among the 10 top ranked models, which accounted together for 0.528 of the Akaike Weight, none of them included three predictor variables. Moreover, the Akaike weight of all models with three variables (0.185) is lower than the Akaike weight of the models including one (0.237) or two variables (0.568). Taken together, these findings suggest that the inclusion of many predictor variables in the models does not increase their explanatory power. In general, models with a Gaussian error distribution had higher Akaike weight than models with a negative binomial or a Poisson error distribution (Table S4). The model including only Cephalothorax (i.e., the best fitted model) explains 20% of the variation in the quantity of sperm stored in the pedipalps (F_1,37_ = 9.269, p = 0.004; R^2^ = 0.200).

In our second approach, we conducted a model selection using Principal Components Analysis (PCA) to reduce the dimensionality of all morphological variables and predict the sperm stored in the pedipalps before copulation. Before the PCA, each morphological variable was standardized, i.e., the cephalothorax width, the residuals of a linear regression between cephalothorax width and body weight, and the areas of the median apophysis area, tegulum, subtegulum, and bulb were centered to their mean and scaled to their standard deviation. The three first principal components (PCs) accumulated 73% of the variance of the six morphological variables (Tables S5-S6). Then, we created models with all the possible combinations of the three PC, with additive and interactive effects between them. For each combination, we constructed models using Gaussian, Poisson and negative binomial (NB) distribution of errors. To select the best combination of predictors to explain the quantity of sperm stored in the pedipalps, we used the Akaike Information Criteria corrected for small samples (AIC_c_).

The results of the model selection are presented in Table S7. We obtained three models with ΔAICc lower than 2: one including PC1, one including PC1+PC3, and one including PC1+PC2. For the sake of simplicity, we used the model including only PC1 as predictor variable of the quantity of sperm stored in the pedipalp. This model explained 17.7% of the variability in the sperm stored (F_1,37_ = 7.95, p = 0.008, R^2^ = 0.177). Thus, the results obtained with the PCA, which synthesizes the variation of the six morphological traits, do not improve our predictive power when compared with the individual morphological traits. Based on this conclusion, we used the results obtained with the individual morphological traits (first approach) in the analyses reported in the main text.

**Table S4.** List of concurrent models including all possible combinations of three or less morphological variables to find the best predictors of the quantity of sperm stored in pedipalps of *Paratrechalea ornata* males before copulation. We also show the number of slopes estimated (*k*) and the Akaike weight (Weight) of each model. The symbol × represents interaction between variables and the symbol + represents additive effects between variables.

| **Predictor variables** | **Distribution** | **ΔAIC_c_** | ***k*** | **Weight** |
| --- | --- | --- | --- | --- |
| Cephalothorax | Gaussian | 0 | 1 | 0.134 |
| Cephalothorax + Subtegulum | Gaussian | 0.70 | 2 | 0.094 |
| Cephalothorax + Bulb | Gaussian | 2.18 | 2 | 0.045 |
| Cephalothorax + Tegulum | Gaussian | 2.28 | 2 | 0.043 |
| Cephalothorax × Apophysis | Gaussian | 2.41 | 3 | 0.040 |
| Cephalothorax + Apophysis | Gaussian | 2.49 | 2 | 0.038 |
| Cephalothorax | NB | 2.49 | 1 | 0.038 |
| Cephalothorax × Tegulum | Gaussian | 2.60 | 3 | 0.036 |
| Cephalothorax × Subtegulum | Gaussian | 2.87 | 3 | 0.032 |
| Cephalothorax + Subtegulum | NB | 3.10 | 2 | 0.028 |
| Cephalothorax + Apophysis + Subtegulum | Gaussian | 3.24 | 3 | 0.026 |
| Cephalothorax + Tegulum + Subtegulum | Gaussian | 3.30 | 3 | 0.026 |
| Cephalothorax + Condition + Subtegulum | Gaussian | 3.34 | 3 | 0.025 |
| Subtegulum | Gaussian | 3.72 | 1 | 0.021 |
| Cephalothorax × Bulb | Gaussian | 3.79 | 3 | 0.020 |
| Apophysis × Subtegulum | Gaussian | 3.90 | 3 | 0.019 |
| Cephalothorax × Apophysis | NB | 4.06 | 3 | 0.017 |
| Tegulum + Subtegulum | Gaussian | 4.14 | 2 | 0.017 |
| Cephalothorax × Tegulum | NB | 4.21 | 3 | 0.016 |
| Tegulum | Gaussian | 4.23 | 1 | 0.016 |
| Cephalothorax + Bulb | NB | 4.49 | 2 | 0.014 |
| Cephalothorax + Tegulum | NB | 4.70 | 2 | 0.013 |
| Cephalothorax + Condition + Bulb | Gaussian | 4.70 | 3 | 0.013 |
| Cephalothorax × Subtegulum | NB | 4.73 | 3 | 0.013 |
| Cephalothorax + Condition + Tegulum | Gaussian | 4.77 | 3 | 0.012 |
| Cephalothorax + Apophysis + Tegulum | Gaussian | 4.87 | 3 | 0.012 |
| Cephalothorax + Condition + Apophysis | Gaussian | 4.97 | 3 | 0.011 |
| Cephalothorax + Apophysis | NB | 4.99 | 2 | 0.011 |
| Tegulum × Subtegulum | Gaussian | 5.36 | 3 | 0.009 |
| Apophysis + Subtegulum | Gaussian | 5.38 | 2 | 0.009 |
| Cephalothorax + Tegulum + Subtegulum | NB | 5.64 | 3 | 0.008 |
| Cephalothorax + Apophysis + Subtegulum | NB | 5.66 | 3 | 0.008 |
| Cephalothorax + Condition + Subtegulum | NB | 5.72 | 3 | 0.008 |
| Apophysis | Gaussian | 5.91 | 1 | 0.007 |
| Bulb | Gaussian | 5.92 | 1 | 0.007 |
| Cephalothorax × Bulb | NB | 5.99 | 3 | 0.007 |
| Apophysis × Subtegulum | NB | 6.13 | 3 | 0.006 |
| Condition + Subtegulum | Gaussian | 6.15 | 2 | 0.006 |
| Null model | Gaussian | 6.40 | 0 | 0.005 |
| Apophysis + Tegulum | Gaussian | 6.55 | 2 | 0.005 |
| Condition + Tegulum | Gaussian | 6.59 | 2 | 0.005 |
| Condition + Tegulum + Subtegulum | Gaussian | 6.77 | 3 | 0.005 |
| Apophysis + Tegulum + Subtegulum | Gaussian | 6.77 | 3 | 0.005 |
| Subtegulum | NB | 6.83 | 1 | 0.004 |
| Cephalothorax + Condition + Bulb | NB | 6.89 | 3 | 0.004 |
| Tegulum + Subtegulum | NB | 6.97 | 2 | 0.004 |
| Cephalothorax + Condition + Tegulum | NB | 7.06 | 3 | 0.004 |
| Tegulum × Subtegulum | NB | 7.09 | 3 | 0.004 |
| Cephalothorax + Apophysis + Tegulum | NB | 7.25 | 3 | 0.004 |
| Tegulum | NB | 7.28 | 1 | 0.004 |
| Cephalothorax + Condition + Apophysis | NB | 7.37 | 3 | 0.003 |
| Condition × Tegulum | Gaussian | 7.72 | 3 | 0.003 |
| Condition + Apophysis + Subtegulum | Gaussian | 8.01 | 3 | 0.002 |
| Condition + Apophysis | Gaussian | 8.14 | 2 | 0.002 |
| Condition + Bulb | Gaussian | 8.20 | 2 | 0.002 |
| Apophysis + Subtegulum | NB | 8.41 | 2 | 0.002 |
| Condition × Subtegulum | Gaussian | 8.55 | 3 | 0.002 |
| Condition | Gaussian | 8.58 | 1 | 0.002 |
| Apophysis × Tegulum | Gaussian | 8.94 | 3 | 0.002 |
| Bulb | NB | 8.97 | 1 | 0.002 |
| Apophysis | NB | 9.01 | 1 | 0.001 |
| Condition + Apophysis + Tegulum | Gaussian | 9.01 | 3 | 0.001 |
| Null model | NB | 9.17 | 0 | 0.001 |
| Condition + Subtegulum | NB | 9.28 | 2 | 0.001 |
| Condition × Apophysis | Gaussian | 9.34 | 3 | 0.001 |
| Condition + Tegulum | NB | 9.56 | 2 | 0.001 |
| Apophysis + Tegulum + Subtegulum | NB | 9.61 | 3 | 0.001 |
| Condition + Tegulum + Subtegulum | NB | 9.61 | 3 | 0.001 |
| Apophysis + Tegulum | NB | 9.64 | 2 | 0.001 |
| Condition × Tegulum | NB | 10.48 | 3 | 0.001 |
| Cephalothorax × Condition × Subtegulum | Gaussian | 10.58 | 7 | 0.001 |
| Condition × Bulb | Gaussian | 10.80 | 3 | 0.001 |
| Condition + Apophysis + Subtegulum | NB | 11.05 | 3 | 0.001 |
| Condition + Apophysis | NB | 11.17 | 2 | 0.001 |
| Cephalothorax × Apophysis × Tegulum | Gaussian | 11.24 | 7 | <0.001 |
| Condition + Bulb | NB | 11.28 | 2 | <0.001 |
| Condition | NB | 11.39 | 1 | <0.001 |
| Condition × Subtegulum | NB | 11.60 | 3 | <0.001 |
| Cephalothorax × Condition × Apophysis | Gaussian | 11.64 | 7 | <0.001 |
| Cephalothorax × Condition × Apophysis | NB | 11.67 | 7 | <0.001 |
| Cephalothorax × Apophysis × Subtegulum | Gaussian | 11.70 | 7 | <0.001 |
| Condition + Apophysis + Tegulum | NB | 12.01 | 3 | <0.001 |
| Apophysis × Tegulum | NB | 12.02 | 3 | <0.001 |
| Condition × Apophysis | NB | 12.17 | 3 | <0.001 |
| Cephalothorax × Apophysis × Tegulum | NB | 12.61 | 7 | <0.001 |
| Cephalothorax × Condition × Subtegulum | NB | 12.81 | 7 | <0.001 |
| Cephalothorax × Condition × Bulb | Gaussian | 12.82 | 7 | <0.001 |
| Cephalothorax × Subtegulum × Tegulum | Gaussian | 13.01 | 7 | <0.001 |
| Cephalothorax × Condition × Tegulum | Gaussian | 13.64 | 7 | <0.001 |
| Cephalothorax × Apophysis × Subtegulum | NB | 13.73 | 7 | <0.001 |
| Condition × Bulb | NB | 13.89 | 3 | <0.001 |
| Cephalothorax × Condition × Bulb | NB | 14.71 | 7 | <0.001 |
| Cephalothorax × Condition × Tegulum | NB | 14.98 | 7 | <0.001 |
| Apophysis × Subtegulum × Tegulum | Gaussian | 15.12 | 7 | <0.001 |
| Cephalothorax × Subtegulum × Tegulum | NB | 15.23 | 7 | <0.001 |
| Condition × Apophysis × Subtegulum | Gaussian | 15.40 | 7 | <0.001 |
| Condition × Apophysis × Tegulum | Gaussian | 15.71 | 7 | <0.001 |
| Condition × Subtegulum × Tegulum | Gaussian | 15.90 | 7 | <0.001 |
| Condition × Apophysis × Tegulum | NB | 16.92 | 7 | <0.001 |
| Apophysis × Tegulum × Subtegulum | NB | 17.05 | 7 | <0.001 |
| Condition × Apophysis × Subtegulum | NB | 17.29 | 7 | <0.001 |
| Condition × Subtegulum × Tegulum | NB | 18.29 | 7 | <0.001 |
| Cephalothorax × Apophysis × Tegulum | Poisson | 72388.34 | 7 | <0.001 |
| Cephalothorax × Condition × Apophysis | Poisson | 72526.73 | 7 | <0.001 |
| Cephalothorax × Condition × Subtegulum | Poisson | 72657.31 | 7 | <0.001 |
| Cephalothorax × Subtegulum × Apophysis | Poisson | 74520.56 | 7 | <0.001 |
| Cephalothorax × Subtegulum × Tegulum | Poisson | 76693.23 | 7 | <0.001 |
| Cephalothorax × Condition × Bulb | Poisson | 76725.25 | 7 | <0.001 |
| Cephalothorax × Condition × Tegulum | Poisson | 77263.50 | 7 | <0.001 |
| Cephalothorax × Tegulum | Poisson | 79964.50 | 3 | <0.001 |
| Cephalothorax × Apophysis | Poisson | 80324.40 | 3 | <0.001 |
| Apophysis × Subtegulum × Tegulum | Poisson | 80928.09 | 7 | <0.001 |
| Cephalothorax × Subtegulum | Poisson | 81267.68 | 3 | <0.001 |
| Condition × Apophysis × Subtegulum | Poisson | 81420.67 | 7 | <0.001 |
| Condition × Apophysis × Tegulum | Poisson | 82207.16 | 7 | <0.001 |
| Condition × Tegulum × Subtegulum | Poisson | 83186.32 | 7 | <0.001 |
| Cephalothorax × Bulb | Poisson | 83288.39 | 3 | <0.001 |
| Cephalothorax + Apophysis + Subtegulum | Poisson | 83386.31 | 3 | <0.001 |
| Cephalothorax + Tegulum + Subtegulum | Poisson | 83526.82 | 3 | <0.001 |
| Cephalothorax + Subtegulum | Poisson | 83661.14 | 2 | <0.001 |
| Cephalothorax + Condition + Subtegulum | Poisson | 83662.41 | 3 | <0.001 |
| Apophysis × Subtegulum | Poisson | 83820.61 | 3 | <0.001 |
| Tegulum × Subtegulum | Poisson | 86296.82 | 3 | <0.001 |
| Cephalothorax + Condition + Bulb | Poisson | 86801.07 | 3 | <0.001 |
| Cephalothorax + Condition + Tegulum | Poisson | 86940.54 | 3 | <0.001 |
| Cephalothorax + Bulb | Poisson | 87077.97 | 2 | <0.001 |
| Cephalothorax + Tegulum + Apophysis | Poisson | 87085.07 | 3 | <0.001 |
| Cephalothorax + Tegulum | Poisson | 87231.37 | 2 | <0.001 |
| Cephalothorax + Condition + Apophysis | Poisson | 87476.85 | 3 | <0.001 |
| Cephalothorax | Poisson | 87804.76 | 1 | <0.001 |
| Cephalothorax + Apophysis | Poisson | 87806.89 | 2 | <0.001 |
| Condition + Tegulum + Subtegulum | Poisson | 91388.96 | 3 | <0.001 |
| Apophysis + Tegulum + Subtegulum | Poisson | 91419.69 | 3 | <0.001 |
| Tegulum + Subtegulum | Poisson | 91420.21 | 2 | <0.001 |
| Condition × Tegulum | Poisson | 93282.83 | 3 | <0.001 |
| Condition + Apophysis + Subtegulum | Poisson | 94414.18 | 3 | <0.001 |
| Apophysis + Subtegulum | Poisson | 94423.34 | 2 | <0.001 |
| Condition × Subtegulum | Poisson | 95481.32 | 3 | <0.001 |
| Condition + Subtegulum | Poisson | 96165.02 | 2 | <0.001 |
| Apophysis × Tegulum | Poisson | 96266.63 | 3 | <0.001 |
| Subtegulum | Poisson | 96288.34 | 1 | <0.001 |
| Condition +Apophysis + Tegulum | Poisson | 96879.58 | 3 | <0.001 |
| Apophysis + Tegulum | Poisson | 97215.05 | 2 | <0.001 |
| Condition + Tegulum | Poisson | 97353.18 | 2 | <0.001 |
| Condition × Apophysis | Poisson | 97570.00 | 3 | <0.001 |
| Tegulum | Poisson | 97626.05 | 1 | <0.001 |
| Condition + Apophysis | Poisson | 101109.72 | 2 | <0.001 |
| Condition × Bulb | Poisson | 101224.92 | 3 | <0.001 |
| Condition + Bulb | Poisson | 101280.73 | 2 | <0.001 |
| Apophysis | Poisson | 101693.42 | 1 | <0.001 |
| Bulb | Poisson | 101801.31 | 1 | <0.001 |
| Condition | Poisson | 108645.73 | 1 | <0.001 |
| Null model | Poisson | 109021.93 | 0 | <0.001 |

**Table S5**. Eigenvectors of the used Principal Components (PC). The values represent the covariance between each morphological variable and the corresponding PC.

| **Morphological variable** | **PC1** | **PC2** | **PC3** |
| --- | --- | --- | --- |
| Bulb | 0.533 | - | 0.287 |
| Subtegulum | 0.320 | 0.223 | -0.625 |
| Tegulum | 0.262 | -0.473 | -0.625 |
| Apophysis | 0.511 | - | 0.111 |
| Cephalothorax | 0.509 | 0.246 | 0.219 |
| Condition | 0.156 | -0.811 | 0.276 |

**Table S6.** Variance explained by the Principal Components Analysis of six morphological traits of the pedipalps of *Paratrechalea ornata* males. For each Principal Component (PC) we present the standard deviation, the proportion of variance explained, and the cumulative proportion of variance.

|  | **PC1** | **PC2** | **PC3** | **PC4** | **PC5** | **PC6** |
| --- | --- | --- | --- | --- | --- | --- |
| Standard deviation | 1.56 | 1.03 | 0.92 | 0.89 | 0.68 | 0.57 |
| Proportion of variance | 0.41 | 0.18 | 0.14 | 0.13 | 0.08 | 0.06 |
| Cumulative proportion | 0.41 | 0.59 | 0.73 | 0.87 | 0.94 | 1.00 |

**Table S7.** List of concurrent models including all the possible combinations of three Principal Components (PC1, PC2, and PC3) to find the best predictors of the quantity of sperm stored in pedipalps of *Paratrechalea ornata* males before copulation. We also show the number of slopes estimated (*k*) and the Akaike weight (Weight) of each model. The symbol × represents interaction between variables and the symbol + represents additive effects between variables.

| **Predictor variables** | **Distribution** | **ΔAIC_c_** | ***k*** | **Weight** |
| --- | --- | --- | --- | --- |
| PC1 | Gaussian | 0 | 1 | 0.265 |
| PC1+PC3 | Gaussian | 1.3 | 2 | 0.138 |
| PC1+PC2 | Gaussian | 1.8 | 2 | 0.109 |
| PC1×PC2 | Gaussian | 2.7 | 3 | 0.069 |
| PC1 | NB | 3 | 1 | 0.059 |
| PC1×PC3 | Gaussian | 3.2 | 3 | 0.055 |
| PC1+PC2+PC3 | Gaussian | 3.9 | 3 | 0.038 |
| PC1+PC3 | NB | 4 | 2 | 0.035 |
| PC1+PC2 | NB | 4.5 | 2 | 0.027 |
| PC1×PC2+PC3 | Gaussian | 4.9 | 4 | 0.023 |
| PC1×PC2 | NB | 5 | 3 | 0.021 |
| PC2×PC3 | Gaussian | 5.2 | 3 | 0.019 |
| NULL | Gaussian | 5.2 | 0 | 0.019 |
| PC1×PC3 | NB | 5.4 | 3 | 0.017 |
| PC1+PC2×PC3 | Gaussian | 5.6 | 4 | 0.016 |
| PC2 | Gaussian | 5.9 | 1 | 0.014 |
| PC1×PC2+PC2×PC3 | Gaussian | 6.5 | 5 | 0.010 |
| PC1+PC2+PC3 | NB | 6.6 | 3 | 0.010 |
| PC1×PC2+PC3 | NB | 7.1 | 4 | 0.008 |
| PC3 | Gaussian | 7.3 | 1 | 0.007 |
| PC1×PC2+PC1×PC3 | Gaussian | 7.9 | 5 | 0.005 |
| NULL | NB | 8 | 0 | 0.005 |
| PC2×PC3 | NB | 8.2 | 3 | 0.005 |
| PC1×PC3+PC2×PC3 | Gaussian | 8.2 | 5 | 0.004 |
| PC2+PC3 | Gaussian | 8.4 | 2 | 0.004 |
| PC1+PC2×PC3 | NB | 8.5 | 4 | 0.003 |
| PC1×PC2+PC2×PC3 | NB | 8.8 | 5 | 0.003 |
| PC2 | NB | 8.8 | 1 | 0.003 |
| PC1×PC2+PC1×PC3+PC2×PC3 | Gaussian | 9.3 | 6 | 0.002 |
| PC1×PC2+PC1×PC3 | NB | 10.1 | 5 | 0.002 |
| PC3 | NB | 10.1 | 1 | 0.001 |
| PC1×PC3+PC2×PC3 | NB | 10.6 | 5 | <0.001 |
| PC2+PC3 | NB | 11.3 | 2 | <0.001 |
| PC1×PC2+PC1×PC3+PC2×PC3 | NB | 11.8 | 6 | <0.001 |
| PC1×PC2×PC3 | Gaussian | 11.9 | 7 | <0.001 |
| PC1×PC2×PC3 | NB | 15 | 7 | <0.001 |
| PC1×PC2×PC3 | Poisson | 77411 | 7 | <0.001 |
| PC1×PC2+PC1×PC3+PC2×PC3 | Poisson | 78226 | 6 | <0.001 |
| PC1×PC2+PC2×PC3 | Poisson | 78877 | 5 | <0.001 |
| PC1×PC2+PC1×PC3 | Poisson | 82140 | 5 | <0.001 |
| PC1×PC2+PC3 | Poisson | 82143 | 4 | <0.001 |
| PC1×PC3+PC2×PC3 | Poisson | 83277 | 5 | <0.001 |
| PC1×PC2 | Poisson | 83716 | 3 | <0.001 |
| PC1+PC2×PC3 | Poisson | 84973 | 4 | <0.001 |
| PC1×PC3 | Poisson | 85012 | 3 | <0.001 |
| PC1+PC2+PC3 | Poisson | 87529 | 3 | <0.001 |
| PC1+PC3 | Poisson | 87595 | 2 | <0.001 |
| PC1+PC2 | Poisson | 88791 | 2 | <0.001 |
| PC1 | Poisson | 90222 | 1 | <0.001 |
| PC2×PC3 | Poisson | 90526 | 3 | <0.001 |
| PC2+PC3 | Poisson | 104675 | 2 | <0.001 |
| PC2 | Poisson | 104758 | 1 | <0.001 |
| PC3 | Poisson | 108133 | 1 | <0.001 |
| NULL | Poisson | 109020 | 0 | <0.001 |

**Table S8.** List of concurrent models to explain three potential benefits of differential allocation in reproductive investment by males of the spider *Paratrechalea ornata*: latency to oviposition, total number of eggs, and mean mass of fertilized eggs. The list includes all possible combinations of the predictor variables (experimental groups and number of flies added to the gift). We used three distributions of errors (Gaussian, Poisson, and gamma) to model the latency to oviposition and the total number of eggs, and two distributions of errors (Gaussian and gamma) to model the mean mass of fertilized eggs. We also show the number of slopes estimated (*k*) and the Akaike weight (Weight) of each model. The symbol × represents interaction between variables and the symbol + represents additive effects between variables.

| **Predictor variables** | **Distribution** | **ΔAIC_c_** | ***k*** | **Weight** |
| --- | --- | --- | --- | --- |
| *Latency to oviposition* |  |  |  |  |
| Experimental groups × Number of flies | Poisson | 0.0 | 3 | 0.467 |
| Experimental groups × Number of flies | Gamma | 1.3 | 3 | 0.244 |
| Experimental groups | Poisson | 2.0 | 1 | 0.171 |
| Experimental groups + Number of flies | Poisson | 4.4 | 2 | 0.052 |
| Experimental groups | Gaussian | 5.6 | 1 | 0.028 |
| Experimental groups × Number of flies | Gaussian | 6.0 | 3 | 0.023 |
| Experimental groups + Number of flies | Gaussian | 8.2 | 2 | 0.008 |
| Experimental groups | Gamma | 9.5 | 1 | 0.004 |
| Experimental groups + Number of flies | Gamma | 9.8 | 2 | 0.004 |
| Null | Gaussian | 26.5 | 0 | <0.001 |
| Number of flies | Gaussian | 28.8 | 1 | <0.001 |
| Null | Gamma | 28.8 | 0 | <0.001 |
| Number of flies | Gamma | 31.1 | 1 | <0.001 |
| Null | Poisson | 39.3 | 0 | <0.001 |
| Number of flies | Poisson | 41.3 | 1 | <0.001 |
| *Total number of eggs* |  |  |  |  |
| Experimental groups + Number of flies | Gaussian | 0.0 | 2 | 0.459 |
| Experimental groups | Gaussian | 1.0 | 1 | 0.276 |
| Experimental groups × Number of flies | Gaussian | 2.7 | 3 | 0.118 |
| Experimental groups + Number of flies | Gamma | 4.6 | 2 | 0.045 |
| Experimental groups | Gamma | 5.2 | 1 | 0.033 |
| Null | Gaussian | 5.5 | 0 | 0.030 |
| Number of flies | Gaussian | 6.5 | 1 | 0.017 |
| Experimental groups × Number of flies | Gamma | 7.3 | 3 | 0.012 |
| Null | Gamma | 8.6 | 0 | 0.006 |
| Number of flies | Gamma | 9.9 | 1 | 0.003 |
| Experimental groups + Number of flies | Poisson | 60.7 | 2 | <0.001 |
| Experimental groups × Number of flies | Poisson | 62.9 | 3 | <0.001 |
| Experimental groups | Poisson | 73.1 | 1 | <0.001 |
| Number of flies | Poisson | 98.4 | 1 | <0.001 |
| Null | Poisson | 103.0 | 0 | <0.001 |
| *Mean mass of fertilized eggs* |  |  |  |  |
| Null | Gamma | 0.0 | 0 | 0.586 |
| Experimental groups | Gamma | 2.4 | 1 | 0.176 |
| Flies | Gamma | 2.4 | 1 | 0.176 |
| Experimental groups + Number of flies | Gamma | 5.0 | 2 | 0.049 |
| Experimental groups × Number of flies | Gamma | 7.7 | 3 | 0.013 |
| Null | Gaussian | 238.3 | 0 | <0.001 |
| Experimental groups | Gaussian | 240.7 | 1 | <0.001 |
| Number of flies | Gaussian | 240.7 | 1 | <0.001 |
| Experimental groups + Number of flies | Gaussian | 243.3 | 2 | <0.001 |
| Experimental groups × Number of flies | Gaussian | 246.0 | 3 | <0.001 |

**References**

Harrell Jr FE. *Regression modeling strategies: with applications to linear models, logistic and ordinal regression, and survival analysis*. Dordrecht: Springer; 2015.

Lessells CM, Boag PT. Unrepeatable repeatabilities: a common mistake. The Auk. 1987;104(1):116-121.
